# A Neural Model of Empathic States in Attachment-Based Psychotherapy

**DOI:** 10.1162/CPSY_a_00006

**Published:** 2017-12-01

**Authors:** David Cittern, Abbas Edalat

**Affiliations:** Algorithmic Human Development, Department of Computing, Imperial College London, London, United Kingdom

**Keywords:** self-attachment, caregiving behavior, self-other pain, compassion, neurobiology

## Abstract

We build on a neuroanatomical model of how empathic states can motivate caregiving behavior, via empathy circuit-driven activation of regions in the hypothalamus and amygdala, which in turn stimulate a mesolimbic–ventral pallidum pathway, by integrating findings related to the perception of pain in self and others. On this basis, we propose a network to capture states of personal distress and (weak and strong forms of) empathic concern, which are particularly relevant for psychotherapists conducting attachment-based interventions. This model is then extended for the case of self-attachment therapy, in which conceptualized components of the self serve as both the source of and target for empathic resonance. In particular, we consider how states of empathic concern involving an other that is perceived as being closely related to the self might enhance the motivation for self-directed bonding (which in turn is proposed to lead the individual toward more compassionate states) in terms of medial prefrontal cortex–mediated activation of these caregiving pathways. We simulate our model computationally and discuss the interplay between the bonding and empathy protocols of the therapy.

## INTRODUCTION

Attachment theory grew out of the work of Bowlby (1969, 1973, 1980), who proposed that to fullfill their basic survival needs, infants had evolved a genetic predisposition to form an attachment relationship with a primary caregiver and that the nature of these early interactions is highly significant with respect to the formation of internal working models of self and other. While sensitive and timely caregiving in response to infant distress and requests for comfort facilitates secure-base exploration and optimal cognitive-emotional neural development and integration; neglectful, inconsistent, and fear-inducing patterns of behavior have been linked to the development of insecure (avoidant, anxious, and disorganized, respectively) attachment schemas. Insecure attachment is thought to leave an individual vulnerable to a variety of psychopathologies (Mikulincer & Shaver, 2012); in the case of disorganization, these include serious disturbances, such as dissociation (Liotti, 1995) and borderline personality disorder (BPD, Carlson, Egeland, & Sroufe, 2009; Fonagy, Target, & Gergely, 2000).

Self-attachment (Edalat, 2015, 2017a, 2017b) is a new, self-administrable, attachment-based psychotherapy that starts from the premise that at the root of many affect dysregulation, mood, and anxiety disorders is a suboptimal attachment experience during early childhood (Mikulincer & Shaver, 2012). Under the self-attachment paradigm, the self of the individual undergoing therapy is conceptualized as comprising two parts: the inner child and the adult self. The inner child corresponds to the emotional self that becomes dominant under times of stress and perceived threat, whereas the adult self corresponds to the more rational self dominant under times of calm and low perceived threat. The therapy aims to re-create the effects of early attachment-based interactions between an infant and a good-enough primary caregiver using instead interactions that are fully internalized, in order to create a secure attachment schema within the individual. This is achieved by means of simulating (e.g., using imagery techniques) the interactions between an infant and a secure caregiver (from both perspectives). These interactions are proposed to naturally stimulate the release of hormones and neurotransmitters like oxytocin (OXT) and dopamine (DA) to encourage neural plasticity and increasingly reduce suboptimal and pathological neural activity that inhibits abilities for self-agency. Because both the inner child and adult self are conceptualized as constituents of the self, the individual can be said to securely “self attach.”

**Figure 1.**
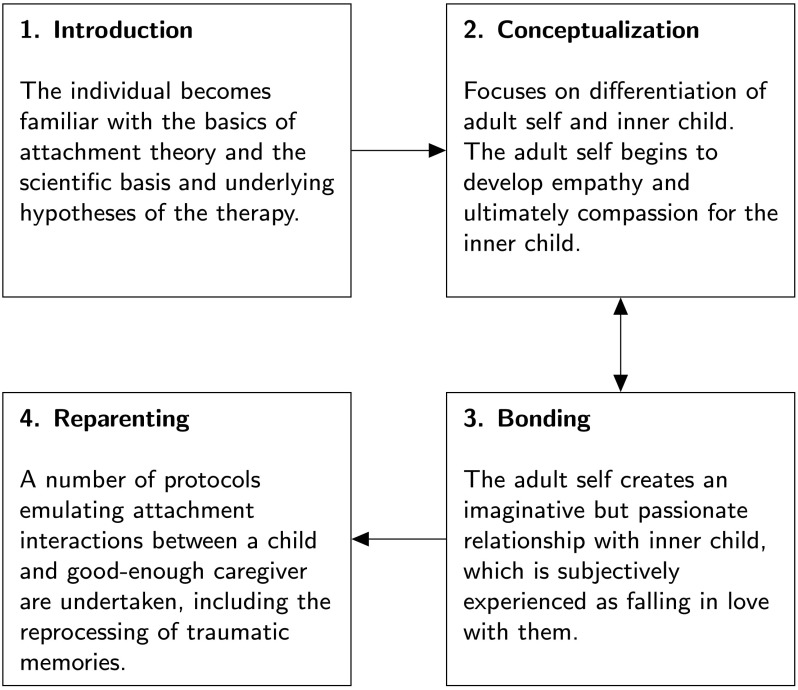
**Four stages of the self-attachment therapeutic process.** See text for further details.

The four stages of the self-attachment therapeutic process (Figure 1) are outlined here (see Edalat, (2015, 2017a, 2017b), for further details). In the first (introductory) stage, the individual becomes familiar with the scientific basis and underlying hypotheses of the therapy, which includes an introduction to attachment theory, and the basics of the (developmental) neurobiology of attachment, love, bond making, and emotion regulation. The aim of this preliminary phase is to provide initial motivation for undertaking the therapy, which requires dedication and self-discipline in terms of time and commitment.

Once this preliminary phase has been completed, the individual can begin to conceptualize the inner child as an entity that is distinct from the adult self, and the adult self can begin to create a relationship with the inner child with a view to establishing empathy and, ultimately, compassion with them. During the second (conceptualization) phase of self-attachment, the individual selects both a positive photograph of his or her childhood (which elicits emotions and memories, such as happiness or contentment) and a negative photograph (e.g., associated with sadness). Several highly structured exercises (termed *protocols*), focused toward these images, are then conducted to conceptualize the inner child as concretely as possible. These protocols include (for example), with closed eyes, trying to visualize the two chosen childhood photos and attempting to imagine that the child that the person was is present and close to the person and that he or she can touch and hold this child. Another protocol, aimed at strengthening the distinction between the adult self and the inner child, involves projection of a negative internal state outward onto an image associated with the inner child. As we discuss later on, we suggest that the undertaking of techniques from existing therapies (e.g., mentalization) might additionally be helpful in strengthening the self–other distinction during this phase.

The third stage of self-attachment is concerned with building an imaginative but passionate affectional bond with the inner child, which is subjectively experienced as falling in love with him or her. First, the adult self adopts the inner child and makes a vow to consistently support and love him or her. Then (from the perspective of the adult self) the individual focuses on the images of the inner child and attempts to bond with him or her, as a basis for creating the internalized attachment relationship. This bonding process is enhanced with the use of activities such as self-massage and positive tactile stimulation (to simulate an embrace) and (overt and/or imagined) song and dance directed toward the inner child, which (as we will expand on) are hypothesized to assist in inducing neural plasticity in key attachment-related neural circuitry (Cittern, 2017; Cittern & Edalat, 2015). Motivation to engage in this bonding may be enhanced with protocols (described later on) involving empathic attunement with the emotional state of the inner child. As we explore, the protocols involved in the second (conceptualization) and third (bonding) phases are closely intertwined, with application of each likely to drive progress in the other, and many of the protocols associated with these phases are carried out in a parallel rather than serial fashion.

The fourth phase of self-attachment therapy involves a number of protocols describing patterns of interaction between the adult self and the inner child that emulate the function of a good-enough parent interacting with a securely attached child, with the aim of minimizing negative emotion and maximizing positive affect. One example is a protocol that involves the reprocessing of painful and traumatic past events: First, the individual closes his or her eyes and recalls a traumatic childhood episode, remembering and reexperiencing in as much detail as possible the associated negative emotions (such as fear or helplessness). Once this state has been recalled, the individual imagines that the adult self quickly and competently intervenes to reduce distress in the inner child, for example, by embracing or vocally reassuring him or her. The aim is for these protocols to become habituated with repetition, so that the individual spontaneously engages with the inner child in ways that alleviate his or her attachment needs.

### Relationship to Existing Therapies

Self-attachment can be regarded as an extension of attachment theory (Edalat, 2017a), but it is also related to and incorporates ideas from a range of existing psychotherapeutic methods (Edalat, 2017b). These include the notion of an “inner child” from transactional analysis (Stewart & Joines, 1987), exposure as in behavioral therapy (Abramowitz, Deacon, & Whiteside, 2012), mentalization and the capacity to understand the emotions and mental states of others as well as those of oneself (Bateman & Fonagy, 2012), schema therapy and reparenting (Young, Klosko, & Weishaar, 2003), object-relations psychodynamic therapy (in a wider sense of the term, in which objects can be impersonal as well as personal, Storr, 1988, p. 150), and cognitive behavioral therapy (which includes techniques to identify the thoughts that induce negative affect and self-distress, challenge these thoughts as irrational or extreme, and replace them with more neutral or positive thoughts and behaviors, Hofmann, 2011). Self-attachment, which can be combined with any well-established therapeutic framework, integrates these techniques into its key and uniquely distinguishing focus of intervention, which is the creation of a fully internalized attachment relationship and affectional bond that emulates the characteristics of a secure infant–parent dyad. Although in its early stages, the results from a small number of initial (uncontrolled) preclinical trials have shown success in tackling chronic anxiety and depression in individuals who had previously (unsuccessfully) engaged with a number of other practices (including cognitive behavioral therapy, psychoanalytic therapy, yoga, mindfulness, and neurofeedback) for long periods over several years (Edalat, 2015).

A particularly closely related concept is security priming (Mikulincer & Shaver, 2007), which involves temporarily activating mental representations relating to the availability of a secure attachment figure to reduce distress and restore positive mood. This is achieved using a variety of techniques involving subliminal (e.g., presentation of pictures suggesting attachment figure availability or of the name of an individual perceived to be a secure attachment figure), visual (e.g., presentation of the face of a secure attachment figure), and imagery (e.g., guided imagery involving availability of an attachment figure) methods.

Also related is compassion-focused therapy (Gilbert, 2009), which involves activities designed to develop compassionate attributes and skills within the individual to improve capabilities for self-compassion and affect regulation. Techniques include the use of imagery, in which the individual imagines himself or herself receiving compassion from an external (not necessarily human) source (Rockliff, Gilbert, McEwan, Lightman, & Glover, 2008). Recent studies have used virtual reality as a medium for switching an individual’s perspective between an adult avatar (resembling the self) and a generic child (Falconer et al., 2016, 2014). While embodied in the adult avatar, the individual administered compassion to the distressed child before switching to the perspective of the child to reexperience himself or herself administering the compassion from this alternative perspective—a practice that resulted in a reduction in measures of depression and self-criticism. In these experiments, a nonrelated and non-self-resembling child avatar was used (although virtual embodiment during the recipient phase would have resulted in some sense of identification with the child). In contrast, rather than being a generic child, the recipient of attachment-based compassion in self-attachment (the inner child) is conceptualized as comprising a part of the self, and there is a focus on the development of a dyadic attachment relationship between the inner child and the adult self. We argue that using this inner child representation, as opposed to a generic and/or nonrelated child, increases the efficacy of the therapy, both from the perspective of primary narcissism (Edalat, 2017a; Freud, 1928/2011) and (as proposed in this article) within the context of optimal representations for inducing empathically motivated caregiving behavior.

In addition, in self-attachment, the individual goes beyond expression of compassion for the inner child by creating an internal affectional bond with that child, emulating the natural bonding between infants and their primary caregivers. This bonding, it has been hypothesized, activates the dopaminergic pathways of the reward system in the brain, providing incentive, hope, and energy for the successful conduct of the therapy (Edalat, 2015). Self-attachment is also differentiated in its use of activities, such as singing, dancing, and self-massage, that are known to increase DA and OXT and reduce cortisol levels (Field, Hernandez-Reif, Diego, Schanberg, & Kuhn, 2005; Jeffries, Fritz, & Braun, 2003; Kleber, Birbaumer, Veit, Trevorrow, & Lotze, 2007; Murcia, Bongard, & Kreutz, 2009). By enhancing positive affects in these ways, individuals practicing self-attachment are better able to counter and contain their negative affects.

Is self-attachment a form of self-therapy, or does it entail interaction with a therapist? The answer is that it can be both. Individuals who are able to conceptualize the inner child and the adult self in themselves may be able to undertake self-attachment as a self-help therapy on their own, in particular if they have already been exposed to some form of psychotherapy before. Others require a standard course of eight sessions in 2–3 months with a trained therapist to learn how to self-administer the self-attachment protocols. In any case, once the individual learns how to self-administer the protocols, he or she is required to practice them regularly by integrating them into their daily routine lifestyle.

### Empathy

As we have outlined, the second (conceptualization) stage of self-attachment involves protocols aiming to empathically attune with the emotional state of an inner child that is in distress, and we have argued that this empathic attunement can assist in generating motivation for the protocols involved in the third stage, concerned with creating an internalized affectional bond between the adult self and the inner child. To examine in detail how these empathic states might generate such motivation in self-attachment, we begin by defining the distinctions between various types of empathic experience and reviewing the role of empathy in attachment-based psychotherapies in general.

Work in empathy distinguishes between a number of related yet distinct phenomena and states: We broadly follow the definitions set out in Gonzalez-Liencres, Shamay-Tsoory, and Brüne (2013), with states of “emotional contagion,” “personal distress,” “emotional empathy,” and “sympathy” being relevant for our initial discussion here. A state of emotional contagion within the self involves a mirroring of the emotional state of another within a context of weak or absent self–other distinction, such that the emotional state of the other is perceived as belonging to the self (and is not necessarily attributed to the other). In cases in which the mirroring is of a negatively valenced emotion, contagion can result in emotional distress within the self (“personal distress”), driving egoistic withdrawal responses in which the self withdraws from its surrounding environment and the stimuli triggering the state to relieve the symptoms of the distress. Similarly to emotional contagion, emotional empathy is a state that arises from a mirroring of the emotional state of another; however, in contrast, this mirroring is accompanied with a strong self–other distinction (i.e., knowledge that this emotional state originates in the other rather than in the self). Because there is this self–other distinction, empathic states involving negatively valenced emotion can drive prosocial motivation aimed at relieving the perceived distress of the other. The term *sympathy* is sometimes loosely used to describe prosocial motivation arising from such an empathic state (we prefer to use the phrase *empathic concern* here).

From an evolutionary perspective, empathic motivation has been argued to have its ultimate roots in selective pressures to care for offspring, identify with in-groups, and exclude out-groups (Zaki, 2014). The mechanisms driving empathic responses may have initially evolved to fulfill parental care (i.e., attachment) responsibilities, while only later being “co-opted” to increase survival prospects of in-groups (Gonzalez-Liencres et al., 2013), suggesting a primacy for attachment in the empathic experience. The infant’s experiences of emotional mimicry and resonance within early attachment experiences have been proposed to underlie the later development of capabilities for empathy toward others (Decety & Meyer, 2008), and a number of studies have shown increased tendencies for self-reported compassionate states and prosocial behavioral responses with increasing attachment security (Mikulincer & Shaver, 2005).

#### Empathy in attachment-based psychotherapy

The role of empathy in psychotherapy dates back to Carl Rogers, who proposed that a continuous effort to empathically attune with the client, along with an unconditional positive regard for him or her, are necessary for therapeutic change to occur (Rogers, 1957). Heinz Kohut’s psychoanalytic self psychology, developed from the 1960s onward, holds that almost all psychopathology is rooted in empathic failure on the part of the parent in childhood, and the therapist serves as an empathic self object to resume development toward maturity (Baker & Baker, 1987; Kohut, 1959). Empathy is now widely accepted as being important for the formation of an effective working relationship between therapist and client. For example, the influential empathy cycle model defines a framework under which communication of the therapist’s ever-strengthening empathic resonance helps to guide the client toward more accurate expression of his or her internal experience (Barrett-Lennard, 1981).

Empathy plays a particularly important role in attachment-based psychotherapies, which view the client–therapist relationship as an attachment bond and encourage the therapist to provide a secure safe haven for the relief of the client’s distress (Obegi, 2008). The therapist uses empathic attunement and contingent communication as tools to help the client explore painful feelings and memories and engages in interactive regulation of the client’s emotion to guide the client toward alternative ways of feeling and acting (Wallin, 2007, p. 196). Thus, to cultivate secure attachment, the client must experience the therapist as being both able and willing to help him or her cope with his or her difficult feelings, and the therapist must engage in a process of interactive regulation of the client’s internal emotional state.

#### Empathically motivated bonding in self-attachment

In self-attachment, empathic resonance with the inner child is proposed as a method for increasing motivation to undertake the protocols related to self-directed bonding (Stage 3), as previously described. The self-attachment empathy protocols involve the individual undergoing the therapy taking the perspective of the adult self while focusing on a negatively emotionally valenced image of his or her younger self (representing the inner child) and attempting to enter into an empathic state with him or her, to cultivate prosocial attitudes, feelings, and states of mind geared toward alleviating distress. Variations of the protocol can involve imagery or virtual reality techniques (Cittern, Edalat, & Ghaznavi, 2017), rather than the focus on an actual image of the younger self, to conceptualize the distressed and assistance-requiring inner child.

The effectiveness of the self-attachment empathy protocols in motivating bonding behavior may vary according to a number of dimensions. We briefly discuss two key factors here but do not consider them in the model that follows (these points are left as considerations for future work). First, as we have highlighted, empathic motivation has been argued to have its ultimate roots in selective pressures for attachment bond formation. Evidence suggests that women (who have typically served as primary caregivers for young infants throughout history and across cultures) have more attuned empathic capabilities with respect to infants relative to men. These traits are believed to have roots more in biological than cultural factors and are again possibly the result of evolutionary pressures (Christov-Moore et al., 2014).

Efficacy of these protocols may also vary according to prior attachment experience. In particular, in BPD (which, as discussed previously, is a condition linked to early dis organized attachment experience), empathic dysfunction may be experienced in the form of hyperreactivity of reflexive systems involved in the sharing of others’ mental states, along with impairments in more deliberative systems involved in perspective taking and the explicit attribution of empathic mental states to the other (Gonzalez-Liencres et al., 2013; Ripoll, Snyder, Steele, & Siever, 2013). Thus, in the case of BPD individuals, care may need to be taken to avoid overwhelming personal distress, and we suggest that the application of these protocols be supplemented with techniques that focus on forming clear mental distinctions between the adult self and the inner child. One such therapy that may be particularly helpful in achieving this aim is mentalization therapy (Bateman & Fonagy, 2012), which involves a joint focus on the client’s subjective inner states to strengthen his or her sense of self and ability to mentalize (i.e., attribute mental and emotional states underlying overt behavior to self and other).

## NEUROSCIENCE OF EMPATHY AND SELF–OTHER PAIN

In this section, we overview evidence relating to the neuroscience of empathic states and self and other perceptions of pain, to lay the groundwork for our computational neural model of personal distress and empathic concern. Imaging studies have uncovered a core empathy-for-pain network (Engen & Singer, 2013) involving the anterior insular (AI; an area involved in a range of emotion-related functions and experiences, including interoceptive awareness, Critchley, Wiens, Rotshtein, Öhman, & Dolan, 2004; Zaki, Davis, & Ochsner, 2012, emotional states induced by imagery or recall, Phan, Wager, Taylor, & Liberzon, 2002, and affective states that arise during social interaction, particularly relating to notions of fairness and cooperation, Lamm & Singer, 2010, and attachment functions, including recognition of the mother’s own infant, Noriuchi, Kikuchi, & Senoo, 2008) and anterior midcingulate cortex (aMCC; a part of the anterior cingulate cortex [ACC], which is involved in a range of social and emotion-related functions, including theory of mind cognition and the perception of fear, Baird et al., 2006, and the detection and appraisal of social exclusion, Kawamoto, Ura, & Nittono, 2015), with lesion-based data furthermore suggesting that the AI is crucial for empathy (Gu, Hof, Friston, & Fan, 2013). The considerable overlap in regions activated during the experiencing of pain oneself, and when perceiving others to be in pain (particularly in the AI and aMCC), has led to a shared-network hypothesis of empathy (Lamm, Decety, & Singer, 2011). A number of factors, including perceived innocence (the degree to which the recipient of the empathic response is deemed to be responsible for his or her fate, Fehse, Silveira, Elvers, & Blautzik, 2015),^1^ closeness (in terms of subjective similarity, or membership of an in-group, Hein, Silani, Preuschoff, Batson, & Singer, 2010), and fairness (i.e., tendency to cooperate, Singer et al., 2006) of the other have been found to modulate the strength of behavioral and neural empathic responses (Engen & Singer, 2013; Numan, 2014).

### Empathically Motivated Caregiving

**Figure 2.**
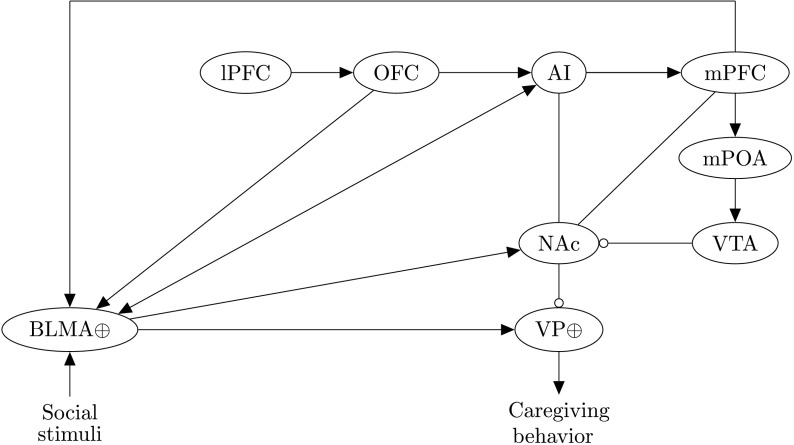
**Numan’s neuroanatomical model for how empathic states can motivate caregiving behavior.** Stimuli enter the BLMA, which projects to the AI to create an empathic state and to the NAc and VP to drive caregiving behavior. The AI projects to the medial prefrontal cortex (including the anterior cingulate), which is proposed to activate a mesolimbic caregiving pathway from the mPOA to the VTA and the NAc, inhibition of which releases the VP from NAc inhibition mediated via BLMA inputs. Projections from lPFC and OFC cortices to the AI and BLMA are additional pathways that can potentiate the caregiving response, as are projections from AI and mPFC to the NAc. Ellipses represent neural populations, arrows are excitatory synapses, and circles are inhibitory synapses (the nature of the connections between AI and NAc and between mPFC and NAc within this context is currently unknown). ⊕ denotes positively valent neurons (i.e., populations that subsequently activate prosocial/approach pathways). From Numan (2014, p. 278). Reprinted with permission from Elsevier.

Numan (2014) has recently proposed a neuroanatomical model (Figure 2) for how empathic states can give rise to caregiving behavior. A large number of studies are cited by him as justification for this model: We overview the most important of those here, along with some additional studies that further support his architecture. We refer to Numan (2014) for a more comprehensive motivation of the model (see also Numan and Young, 2016, and Numan, 2017, for details on underlying circuits involved in parental caregiving behavior).

Under the model, projections from the basolateral and basomedial nuclei of the amygdala (basolateral and basomedial amygdala [BLMA]; a major input region for social stimuli, which has long been associated with Pavlovian fear conditioning, Feinstein, Adolphs, Damasio, & Tranel, 2011, but is now also known to be involved in goal-directed behavior and to respond to both learned and innately rewarding stimuli, Jenison, Rangel, Oya, Kawasaki, & Howard, 2011; Morrison & Salzman, 2010) to the AI are proposed to facilitate the creation of the shared empathic state (Hurlemann et al., 2010). It is proposed that, when AI activation levels are high enough, this region stimulates the medial prefrontal cortex (mPFC; which includes the ACC, and thus aMCC, in Numan’s definition), which in turn activates prosocial pathways. Supporting this claim, empathy-related activity in the AI and mPFC has been found in response to viewing an unfairly treated other (in the form of exclusion from a ball-tossing game) with activity correlating with subsequent (spontaneous) prosocial behavior (Masten, Morelli, & Eisenberger, 2011; similar results were reported by Mathur, Harada, Lipke, & Chiao, 2010, in response to viewing the suffering of another perceived as being in-group).

In particular, it is proposed by Numan that more ventral parts of the mPFC might be activated most strongly during the empathic concern response. There is evidence to suggest that the mPFC encodes a ventral–dorsal gradient for self–other reflection (Denny, Kober, Wager, & Ochsner, 2012) that is also sensitive to self-relatedness of the other, with reflection on others perceived to have high degrees of self-relatedness (e.g., in terms of similarity, familiarity, and closeness) correlating with more ventral mPFC activation, and reflection on non-self-related stimuli (e.g., a publicly known but personally unknown other) correlating with more dorsal mPFC activation, and that this gradient is sensitive within the context of empathy. Using fMRI, Meyer et al. (2012) investigated the neural correlates of individuals viewing the social exclusion of either a friend or stranger and found that observation of the friend was associated with relatively higher activation in the ventromedial prefrontal cortex (vmPFC). There is also evidence to suggest that more ventral parts of the mPFC activate in response to a perceived innocence of the other within the context of empathy: Fehse et al. (2015) found higher self-reported empathic concern, along with elevated activation of (more ventral parts of) the mPFC (with reported coordinates corresponding to a region near to the ACC), in response to stimuli depicting individuals who had experienced an unfortunate fate but were described as being innocent rather than responsible for their situation. These studies are consistent with Numan’s proposal that more ventral parts of the mPFC are associated with relatively high subsequent activation in caregiving pathways in response to empathic resonance, according to a ventral–dorsal encoding of self and other representations in this area. An alternative perspective on mPFC functioning (Nicolle et al., 2012) proposes that activity in more ventral parts of the mPFC is associated with agent-independent choice value for executed behavior (i.e., value for the self when the self chooses on behalf of the self, or value for the other when the self chooses on behalf of the other), whereas activity in more dorsal areas of the mPFC is associated with modeled value (i.e., value for the other when the self chooses on behalf of the self, or value for the self when the self chooses on behalf of the other). According to this view, we might similarly expect value for empathic concern behavior (toward an other perceived as being in distress) as executed by the self to be represented by activity in vmPFC.

The mPFC is proposed to initiate caregiving behavior via activation of the ventral pallidum (VP) along both a stimulatory mPFC–BLMA–VP pathway and a disinhibitory mPFC–medial preoptic area (mPOA)–ventral tegmental area (VTA)–nucleus accumbens (NAc)–VP pathway (with inhibition of the NAc serving to release the VP from BLMA-mediated inhibition, thus potentiating caregiving behavior). VP projections to midbrain locomotor regions have long been proposed to be involved in the translation of limbic motivation signals into motor output (Brudzynski, Wu, & Mogenson, 1993; Jordan, 1998; Mogenson, 1987), and increasing activation in ventromedial parts of the VP (in response to NAc shell-mediated disinhibition) have been associated with goal-directed behavior (Numan, 2014; Root, 2013, pp. 24–25).

The first of these pathways (mPOA–VTA–NAc–VP) has been identified as crucial for the onset and maintenance of maternal and caregiving behavior based on a large body of animal (lesion) studies, and it is proposed that these same pathways also underlie the motivational aspects of empathic concern in humans (Numan, 2014). In particular, in animals, hormones and neuropeptides (including OXT) act on the mPOA (a part of the hypothalamus, which is in general an integrative region that brings together a range of inputs relating to the internal environment; compares them to setpoints [ideal ranges], and activates autonomic, endocrine, and behavioral responses to maintain the internal state within these ranges; Saper & Lowell, 2014), resulting in projections from this region to the mesolimbic DA system in response to infant stimuli. The subsequent DA release from the VTA serves to inhibit the NAc, which releases the VP from inhibition and allows it to be responsive to infant stimuli-mediated projections from the BLMA. In animal studies, activation of this circuit has been found to result in motivated responses that attract a mother to her young, and disruption of projections from the mPOA to the mesolimbic pathway halt this attraction and associated caregiving behavior (Numan, 2014; Numan et al., 2005).

Because inactivation of mPFC projections to mPOA are also known to disrupt pup retrieval in rats (Numan, 2014, p. 191), the mPFC is suggested as a crucial link between areas involved in the formation of empathic states and the disinhibitory mPOA–mesolimbic DA pathway identified as being crucially involved in caregiving behavior. A number of studies are presented as evidence for involvement of this same pathway in prosocial and caregiving behaviors arising from empathy in humans, one of which is the investigation by Moll et al. (2006) that used fMRI to uncover the correlates of prosocial behavior related to giving (in the form of a monetary donation) and receiving reward. While both the receiving and giving of reward correlated with activity in the VTA and NAc, donation correlated additionally with activation in the preoptic area and Brodmann area (BA) 25 (a part of the vmPFC) and increased activation in the NAc. This suggests that the vmPFC and preoptic area might interact with the mesolimbic DA system within the context of prosocial acts that are interpreted as being rewarding to the recipient, as is the case in empathic concern responses. Interactions between the preoptic area and the mesolimbic DA system have furthermore been observed during the simulation of prosocial acts: In the fMRI study conducted by Decety and Porges (2011), participants viewed scenes including those involving individuals easing the pain of others and were then asked to mentally simulate being the performer of such acts. In simulatory but not viewing scenarios, the authors found increased activation in the preoptic area and NAc, plus increased functional connectivity between the amygdala and NAc and VP. Using simulation theory (Hesslow, 2002), the authors argued that actual overt prosocial action would be expected to activate the same pathways as were found to be activated during these simulations. This suggests that activation of the same mPOA–mesolimbic DA pathway might drive both overt and imagined empathic concern responses.

The BLMA is anatomically positioned to relay the olfactory and somatic sensory inputs from infants (important for maternal and caregiving behavior) to the NAc and VP, and suppression of BLMA activity and its input to the VP have been found to disrupt maternal and caregiving behavior in rats (Numan et al., 2010). Within the context of empathic concern responses in humans, it is suggested in the model that projections from the mPFC to the BLMA are a significant pathway by which the type of social stimuli that can gain access to positively valent neurons in the BLMA and VP are regulated, allowing in-group members priority access to prosocial circuits. Finally, it is suggested that the lateral prefrontal cortex (lPFC) and the orbitofrontal cortex (OFC) might also be involved in empathic concern responses in the form of cognitive modulatory influences via projections to the BLMA and AI. Later on, we will consider in particular a possible role of the medial orbitofrontal cortex (mOFC) within this framework related to findings from the neuroscience of compassion and the hypothesized effects of the self-attachment bonding protocols.

### Neural Correlates of Self and Other Pain Attribution

To extend Numan’s model to additionally consider a state of personal distress (which behaviorally has been found to induce egoistic withdrawal as opposed to caregiving behavior), we consider now the neural correlates of self and other pain attribution. Singer et al. (2004) used fMRI to asses brain activity while volunteers either experienced a painful stimulus (electrode) themselves or received a cue indicating that their loved one (present in the same room) was receiving a similar painful stimulus. Their experiment followed on from a number of previous studies that had consistently shown activation in a pain network spanning the secondary somatosensory cortex (an area thought to be involved in the processing and integration of both painful and nonpainful somatosensory stimuli that are salient for higher order functions such as memory and attention, Chen et al., 2008), the insular cortex, the ACC, the cerebellum and supplementary motor areas (which are involved in movement, motor control, and adaptation; Glickstein, 2007), and (less consistently) the thalamus (which relays and controls the flow of information to the cortex; Sherman & Guillery, 2002) and primary somatosensory cortex in response to painful noxious stimuli. The authors found that areas including the bilateral AI, rostral (perigenual) ACC, brainstem, and cerebellum activated in both self and other pain conditions, whereas activity in the left posterior insular (PI)/secondary somatosensory cortex and right mid insular (MI) areas (areas otherwise implicated in the interoceptive, Craig, 2011, and sensory-discriminatory, Pavuluri & May, 2015, aspects of pain), caudal ACC, and sensori motor cortex (an area that is thought to be involved in both imagery and execution of motor function; Stippich, Ochmann, & Sartor, 2002), comprising a pain network that has commonly been found to activate in response to painful noxious stimuli, was specific to the self pain condition. The authors concluded that empathizing with others in pain does not involve activation of the whole of the pain network and that empathy is mediated by areas involved in representing the affective (but not sensory) aspects of pain.

Similarly, Zaki, Ochsner, Hanelin, Wager, and Mackey (2007) conducted an fMRI study to determine the neural correlates of pain when experienced by the self and contrasted these with the correlates of pain perceived to be experienced by another. During the self-pain condition, a thermal noxious stimulus was administered to the individual, while under the other-pain condition, participants watched videos of other people receiving pain-inducing injuries. On the basis of these data, contrast (Ochsner et al., 2008) and functional connectivity (Zaki et al., 2007) analyses were performed. In the contrast analysis (which looked at relative activation levels), a number of regions were found to have common activation during both self and other pain conditions. These regions include the aMCC and AI (regions previously implicated in the shared-network hypothesis of empathy), along with the middle frontal and premotor gyri and the dorsal thalamus. The AI, PI, and middle frontal gyrus were found to be more activated for self pain as opposed to other pain, whereas in contrast, greater activation was found in regions including the precuneus (an area that has been implicated in functions including the experience of a sense of agency, Cavanna & Trimble, 2006, and the recall of autobiographical memories involving familiar others, Maddock, Garrett, & Buonocore, 2001), OFC (which is involved in a broad range of social-emotion processing and regulatory tasks, including the inhibition of socially inappropriate and impulsive behaviors, Beer, John, Scabini, & Knight, 2006, and the rapid response in the parent to a range of infant cues, Parsons, Stark, Young, Stein, & Kringelbach, 2013), and amygdala for other as opposed to self pain. The functional connectivity analysis revealed distinct circuits involved in the perception of pain in the self and other. While the AI and aMCC were found to be functionally connected to each other during both self and other pain, these regions showed increased functional connectivity with more posterior (mid) areas of the insular during self pain, along with the periaqueductal gray (which is involved in pain modulation and sensations associated with aversive emotions; Mai & Paxinos, 2011, p. 367) and areas in the midbrain. In contrast, during other pain, these regions were more functionally connected with a network comprising mPFC, precuneus, posterior cingulate cortex (PCC; a region that, similarly to the precuneus, is thought to be involved in the recall of autobiographical memories involving familiar others; Maddock et al., 2001), and superior temporal sulcus (STS), which has been implicated in a variety of social processes including theory of mind; Beauchamp, 2015).

## A MODEL OF PERSONAL DISTRESS AND EMPATHIC CONCERN

**Figure 3.**
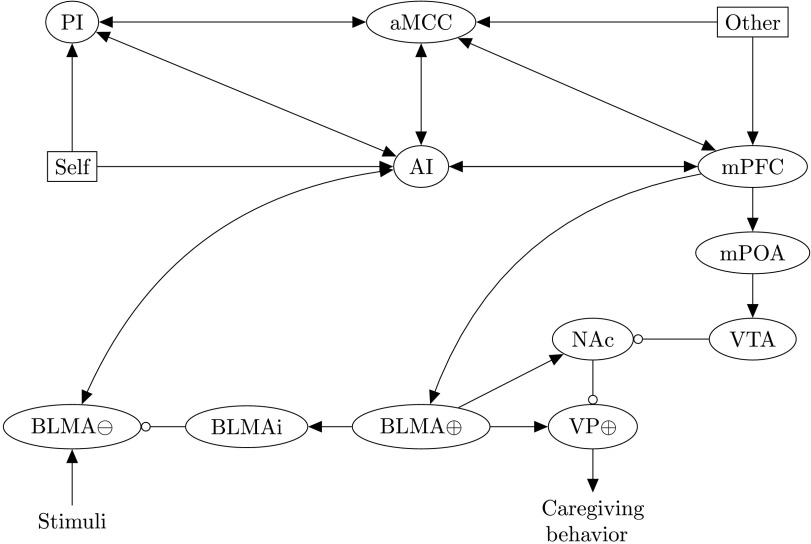
**Model capturing how self-other distinction can mediate empathically motivated caregiving behavior in response to pain-inducing stimuli.** The model is based on an expanded version of Numan’s framework (Figure 2) to incorporate data on the self–other distinction within the context of pain, to account for the distinct states of personal distress, and (weak and strong) empathic concern. During the self pain condition (associated with personal distress), the PI and BLMA⊖, associated with egoistic withdrawal/avoidance pathways, activate the AI and aMCC. During the other pain conditions (associated with empathic concern), BLMA⊕ are activated to potentiate caregiving behavior and inhibit BLMA⊖ avoidance circuitry. The strong empathic concern state (involving close other representations) is associated with stronger mPFC activation of prosocial pathways compared to a weak empathic concern state (associated with relatively more distant other representations). Ellipses represent neural populations, arrows are excitatory connections, and circles are inhibitory connections (intragroup excitatory/inhibitory connections not shown). Rectangles (Self, Other) are points of current injection, proposed to represent activity in distinct neural networks involved in the perception of self and other pain (see text for details). ⊕ denotes positively valent neurons (i.e., populations that subsequently activate prosocial/approach pathways) and ⊖ negatively valent neurons (that subsequently activate withdrawal/avoidance pathways).

As discussed previously, empathy plays an important role in psychotherapy and particularly in attachment-based therapies. In this section, we propose (and simulate) a computational neural model to distinguish between states of personal distress and (weak and strong forms of) empathic concern. An understanding of how representations of self and other might medi ate between such states is important, because therapists and other health workers engaging in empathic resonance without a sufficiently strong self–other distinction can become susceptible to secondary trauma (the secondhand exposure to traumatic events), burnout (overwhelming emotional exhaustion), alexithymia, and self-focused emotional distress (Gleichgerrcht & Decety, 2013; Wagaman, Geiger, Shockley, & Segal, 2015; Zenasni, Boujut, Woerner, & Sultan, 2012). The model developed here will also serve as a basis for examining the role of empathy in self-attachment therapy in the following section. The overall architecture is shown in Figure 3.

Here we have attempted to extract key findings from the studies (detailed previously) on self and other pain networks to model states of personal distress and empathic concern. In particular, we consider two points of current injection (self and other). According to Zaki’s model, self pain involves increased activation in both the AI and PI: To capture this, during the self pain condition, we inject current into neurons in both of these regions. These injections to the insular might feasibly represent projections along a midbrain–periaqueductal gray pathway, because these regions are both anatomically and (during self pain) functionally connected to this region, and/or via a pathway involving the thalamus and hypothalamus, which showed preferential activation for self- as opposed to other-referential processing in a separate meta-analysis (Zaki & Ochsner, 2011, p. 21) and have strong connectivity with this region.

During the other pain condition, we input current to neurons in the aMCC and mPFC. Input to the aMCC may represent inputs from the precuneus in Zaki’s other pain network, because this region was found to be both more active, and more connected to the aMCC, during other pain, and there are known anatomical connections between these regions. Input to the mPFC during the other pain condition represents activation of neurons preferentially encoding other-referential processing, and we propose that activation of a subset of these neurons encoding close others will most strongly drive activation in the caregiving pathways described by Numan. This current injection might also represent increased activation in the precuneus, which is anatomically connected with the mPFC via the PCC.

### Desired Network States and Simulation Phases

Here we describe three network states that are modeled in distinct phases of our simulation. The network is simulated at 1 ms granularity, with discrete iterations *t* = 1,2,…, 30,000, giving a total of 30 s simulation time. We split our simulation into three phases of equal length *p* = 10,000, where each phase corresponds to one of the three network states.

At each iteration, current is injected into neurons in the negatively valenced neurons of the basolateral and basomedial nuclei of the amygdala (BLMA⊖; representing the pres ence of a stimulus of an individual in distress), the AI and PI (representing input from the self-pain network), and the aMCC and mPFC (representing input from the other pain network). Note that ⊕ represents positively valent neurons (i.e., populations that subsequently activate prosocial/approach pathways) and ⊖ negatively valent neurons (which subsequently activate withdrawal/avoidance pathways). We define three values representing different proportional levels of stimulation: *L* = 0.05 (“low”), *M* = 0.1 (“medium”), and *H* = 0.15 (“high”). For each of these five neural groups *G* ∈ {BLMA ⊖, AI, PI, aMCC, mPFC}, we now designate a subset of neurons *g*(*G*) ⊂ *G* that can receive current injection, where |*g*(*G*)| = |*G*|* *H*. For BLMA ⊖, AI, PI, aMCC, the neurons composing this subset *g*(*G*) are chosen randomly according to a uniform distribution U.

Recall that the mPFC is believed to encode self–other representations along a ventral–dorsal gradient: We have connected neurons in this region to Numan’s caregiving pathway accordingly (see Table A.5). In particular, the mPFC has been connected to the mPOA and positively valenced neurons of the basolateral and basomedial nuclei of the amygdala (BLMA⊕) according to a negative-binomial distribution parameterized by *r* = 7 and *p* = 0.0025, which is intended to model this ventral–dorsal gradient for self–other representations in the mPFC (i.e., neurons representing self or distant other will be sampled with relatively low probability). In the simulations that follow, we consider two distinct target populations in the mPFC for neural activity in Zaki’s other pain network: a first population (encoding close other representations) that is sampled according to this negative-binomial distribution (i.e., with *r* = 7 and *p* = 0.0025) and a second population (encoding more distant other representations) that is sampled according to a negative-binomial distribution with *r* = 14 and *p* = 0.035 (see Figure A.1).

We have a total of five subgroups *g* of neurons that can potentially receive external current on each iteration. At each time step *t*, before each current injection, each of these neural subgroups *g* is perturbed by 10*%* (by random-uniformly switching 10*%* of neurons designated to receive current injection with neurons that previously were not). This perturbation results in *g*_*t*_(*G*), which defines the subset of neurons in neural group *G* that can potentially receive current injections at time step *t*. This subset is then used to determine the final subset of neurons *c*_*t*_(*G*) ⊆ *g*_*t*_(*G*) that actually receive current at time step *t*, chosen according to a random-uniform distribution and varied according to which phase the simulation is currently in. For simplicity, we use an amplitude 90 mA for all current injections and capture the different network states by varying *c*_*t*_(*G*) for all *G* across the phases.

At all iterations *t* (i.e., across all phases), we inject currents into a random subset *c*_*t*_(BLMA ⊖) ⊆*g*_*t*_(BLMA ⊖) of BLMA⊖ neurons, where the size of this subset |*c*_*t*_(BLMA⊖)— ∼U{—BLMA ⊖—* *M*, —BLMA ⊖— * *H* } is a uniformly distributed integer in the interval [—BLMA ⊖— * *M*, —BLMA ⊖— * *H*]. This current injection into the BLMA⊖ represents high levels of negatively valent input stimulation across the whole simulation.

#### Personal distress

The first state that we want to capture is that of personal distress. In accordance with the findings of Ochsner et al. (2008) described previously, AI and PI activations should be relatively high for this state, and because personal distress is associated with withdrawal rather than prosocial behavior, we should additionally have low activity in the VP (which drives caregiving behavior). Relatively high activity in the BLMA ⊖–AI–PI network, along with low activity in the VP, thus defines a network state corresponding to personal distress.

In accordance with the above, during this phase of the simulation, we inject current at a high level into both the AI and PI (in addition to current injections into the BLMA ⊖). This corresponds to stimulating a random subset *c*_*t*_(AI) ⊆ *g*_*t*_(AI) of AI neurons (with $\left|c_{t}(\text{AI})|∼U\{|\text{AI}|*M,|\text{AI}|*H\right\}$) and a random subset *c*_*t*_(PI) ⊆ *g*_*t*_(PI) of PI neurons (with $\left|c_{t}(\text{PI})|∼U\{|\text{PI}|*M,|\text{PI}|*H\right\}$. During this phase, we also inject current at a low level into the aMCC and mPFC (to represent low levels of activity in the other pain network, i.e., a weak self–other distinction). This corresponds to stimulating random neuron subsets *c*_*t*_(aMCC) ⊆ *g*_*t*_(aMCC) (with $\left|c_{t}(\text{aMCC})|∼U\{|\text{aMCC}|*L,|\text{aMCC}|*M\right\}$) and *c*_*t*_(mPFC) ⊆ *g*_*t*_(mPFC) (with $\left|c_{t}(\text{mPFC})|∼U\{|\text{mPFC}|*L,|\text{mPFC}|*M\right\}$). Current is injected into mPFC neural populations encoding a close other representation (Figure A.1).

#### Weak empathic concern

The second state that we want to capture (which we call *weak empathic concern*) is that of an empathic state with a relatively strong self–other distinction (compared to the personal distress state), but with an other stimulus that is encoded as being a relatively distant other. Despite this distant other encoding, the weak empathy state should nonetheless potentially be sufficient for the motivation of caregiving behavior. In this state, we should again have activation in the BLMA⊖ (as a result of input stimuli representing a distressed other) and in the AI and aMCC (which form core parts of empathy circuitry). In accordance with the findings of Ochsner et al. (2008), AI and PI activations should be relatively low compared to a personal distress state, while aMCC should be roughly the same. Activity in mPFC neurons encoding distant other representations should trigger activity in the BLMA⊕–VP and mPOA–VTA–NAc–VP pathways that facilitate caregiving behavior (at a level that is relatively high compared to the personal distress state but relatively low compared to the strong empathic concern state considered next).

During the second phase of the simulation, in addition to current injections into the BLMA⊖, we thus inject current at a low level into both the AI and PI. This corresponds to stimulating a random subset *c*_*t*_(AI) ⊆ *g*_*t*_(AI) of AI neurons (with $\left|c_{t}(\text{AI})|∼U\{|\text{AI}|*L,|\text{AI}|*M\right\}$) and a random subset *c*_*t*_(PI) ⊆ *g*_*t*_(PI) of PI neurons (with $\left|c_{t}(\text{PI})|∼U\{|\text{PI}|*L,|\text{PI}|*M\right\}$. We also inject current at a high level into the aMCC and mPFC (to represent high levels of activity in the other pain network). This corresponds to stimulating random neuron subsets *c*_*t*_(aMCC) ⊆ *g*_*t*_(aMCC) (with $\left|c_{t}(\text{aMCC})|∼U\{|\text{aMCC}|*M,|\text{aMCC}|*H\right\}$) and *c*_*t*_(mPFC) ⊆ *g*_*t*_(mPFC) (with $\left|c_{t}(\text{mPFC})|∼U\{|\text{mPFC}|*M,|\text{mPFC}|*H\right\}$). Current is injected into mPFC neurons encoding more distant other representations (Figure A.1).

#### Strong empathic concern

The third state that we want to capture (which we call *strong empathic concern*) is that of an empathic state with a strong self–other distinction and a target that is now perceived to be a close other. This close other encoding should result in a relatively high level of motivation for caregiving behavior compared to the weak empathic concern state. Activation levels across neural populations should thus be similar as for the weak empathy state, except that we should now expect relatively high activity in the BLMA⊕–VP and mPOA–VTA–NAc–VP pathways.

Current injections for the third phase of the simulation capturing strong empathic concern are the same as for the weak empathic concern state, except that current is now injected into mPFC neurons encoding close other representations rather than distant other representations (Figure A.1).

### Simulation Results

Here we describe results of simulations of our network over the three phases described in the previous section. In brief (and as covered previously), the first phase, for time steps *t* ∈ (0,10] s, corresponds to a personal distress response, during which activation inputs from the self and other pain networks are high and low, respectively (with inputs from the other pain network targeting close other mPFC representations). The second phase (*t* ∈ (10,20] s) corresponds to a weak empathic concern state (i.e., a state in which the other’s emotional state is mirrored, with the other being perceived as a relatively distant other). Input from the self pain network is low, and from the other pain network is high, and the other pain network stimulates mPFC neurons encoding close other representations, leading to a relatively high level of caregiving behavior compared to the personal distress state. The third phase (*t* ∈ (20,30] s) corresponds to a strong empathic concern state, with strong self–other distinction and the other perceived as a close other. During this phase, input from the self pain network is low, while input from the other pain network is high (and targets mPFC neurons encoding close other representations), such that relatively high amounts of caregiving behavior result as compared to the weak empathy state. Our simulations are implemented using the CARLsim 3.1 framework (Beyeler, Carlson, Chou, Dutt, & Krichmar, 2015) with Izhikevich neurons and a network topology as described in Cittern and Edalat (2017, supplement), and results presented are representative of a typical simulation run.

**Figure 4.**
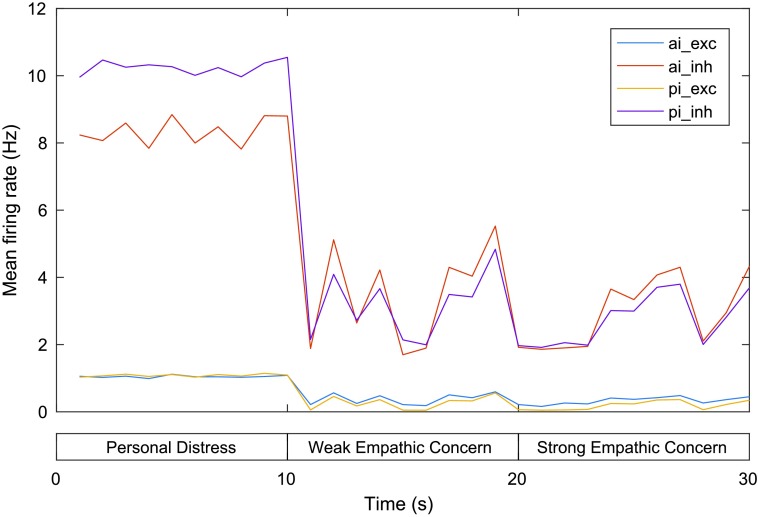
**Mean firing rates for the AI and PI in the model of personal distress and empathic concern.** exc = excitatory neurons, inh = inhibitory neurons. See text for details.

Figure 4 gives the mean firing rate (MFR) for neurons in the excitatory and inhibitory AI and PI neural groups. The chart shows that the MFR of AI and PI neurons is highest during the first phase (personal distress) and drop significantly relative to this during the final two phases (corresponding to weak and strong empathic concern). Excitatory/inhibitory AI neurons drop from a MFR of 1.08/8.80 Hz at 10 s (end of the first phase and beginning of the second phase) to 0.22/1.92 Hz at 20 s (the end of the second phase) and rise again slightly to 0.45/4.33 Hz at 30 s (end of the third phase), while excitatory/inhibitory PI neurons drop from a MFR of 1.09/10.55 Hz at 10 s to 0.06/1.97 Hz at 20 s and rise slightly to 0.34/3.69 Hz at 30 s. These patterns correspond directly with the results of the experiments (described previously) regarding activation in these regions for pain perceived to be experienced by the self relative to another.

**Figure 5.**
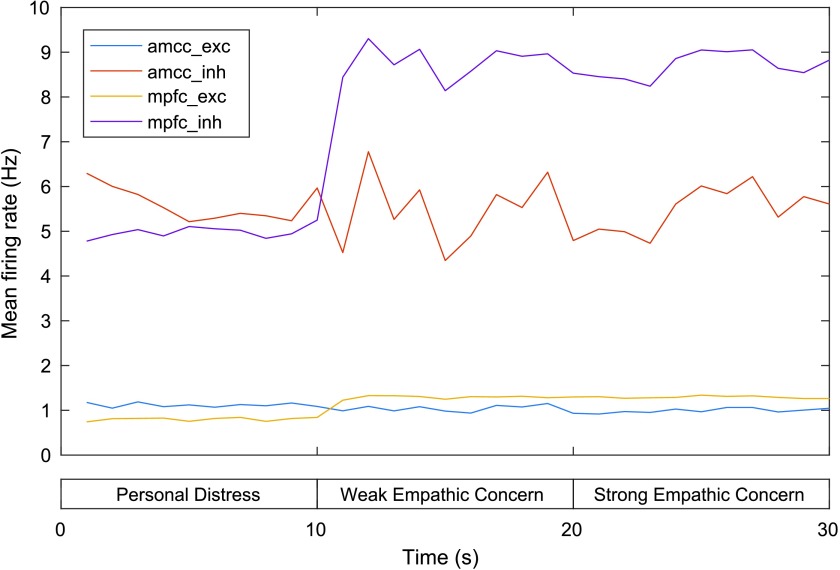
**Mean firing rates for the aMCC and mPFC in the model of personal distress and empathic concern.** exc = excitatory neurons, inh = inhibitory neurons. See text for details.

MFR for neurons in the excitatory and inhibitory aMCC and mPFC groups are shown in Figure 5. The aMCC MFR is relatively flat across the three phases: The MFR for excitatory/ inhibitory aMCC is 1.09/5.97 Hz at 10 s, 0.93/4.79 Hz at 20 s, and 1.05/5.61 Hz at 30 s. This relative stability is in accordance with the findings of the experiments (described previously) regarding attribution of pain perception, which did not find significant differences in activation of the aMCC across self and other pain paradigms. On the other hand, the MFR of the mPFC is slightly higher in the second and third phases compared to the first phase (0.84/5.25 Hz at 10 s, 1.30/8.53 at 20 s, and 1.26/8.82 at 30 s). This coincides with increased stimulation of neurons encoding distant and close other representations in this region by the other pain network during the second and third phases (the mPFC is not directly stimulated with external current during the self pain condition).

**Figure 6.**
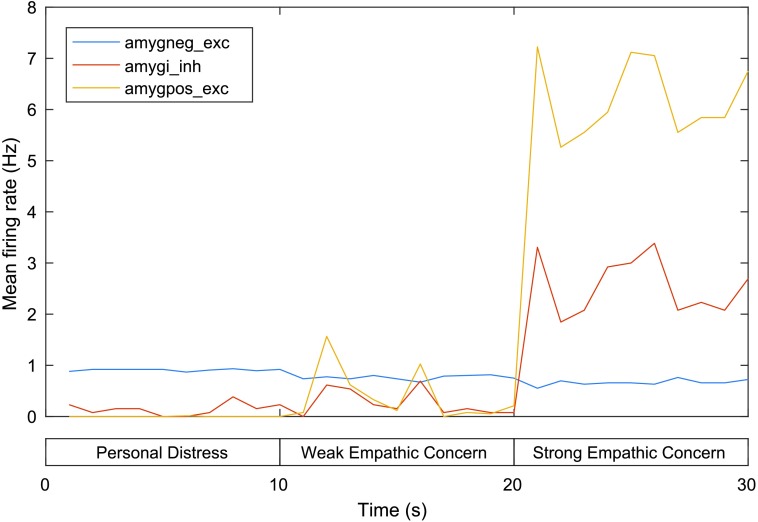
**Mean firing rates for the three BLMA neural populations in the model of personal distress and empathic concern.** exc = excitatory neurons, inh = inhibitory neurons. See text for details.

Figure 6 shows the MFR for the three BLMA neural populations. The BLMA⊖, which receives a steady input current across all three phases (corresponding to client stimulus input), has a MFR that is relatively flat across all three phases, dropping slightly for the second and third phases relative to the first (0.92 Hz at 10 s, 0.75 at 20 s, 0.72 at 30 s). This drop during the third phase coincides with an increase in MFR of the BLMA⊕ (from 0.21 Hz at 20 s to 6.75 Hz at 30 s), whose neurons are stimulated by the mPFC and excite the inhibitory interneurons of the basolateral and basomedial nuclei of the amygdala (BLMAi).

**Figure 7.**
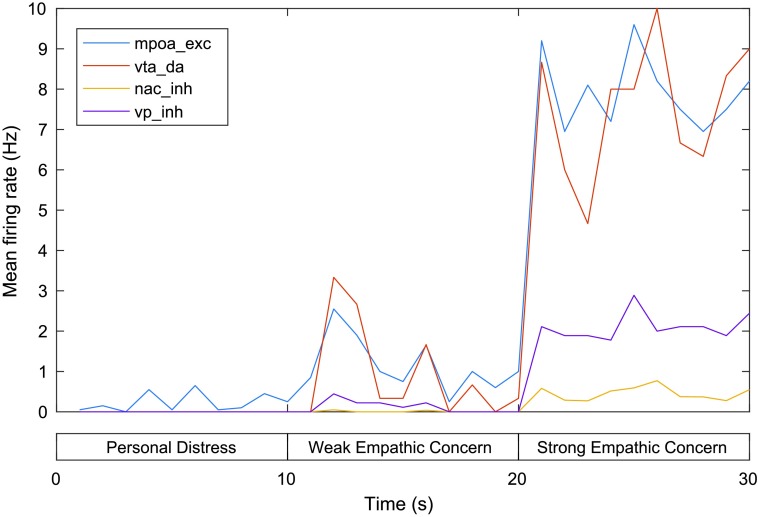
**Mean firing rates for the mPOA, VTA, NAc, and VP in the model of personal distress and empathic concern.** exc = excitatory neurons, inh = inhibitory neurons, da = dopaminergic neurons. See text for details.

Finally, MFR for the mPOA, VTA, NAc, and VP are shown in Figure 7. Activity in the mPOA, VTA, and NAc is relatively low during the first phase and rises significantly during the second phase (with a stronger self–other distinction and perception of the other as a relatively distant other) and further across the third phase (in which the other is instead encoded as a close-other). These firing patterns are reflected in firing in the VP, with relatively low MFR in the VP during the first phase but increased firing during the second phase and higher firing yet during the third phase (with MFR 2.44 Hz at 30 s). Firing of the VP (which facilitates caregiving behavior) is thus low during personal distress, increased for the weak empathic concern state, and highest for strong empathic concern.

In summary, our model replicates data on relative AI and PI activation across self and other pain states (with relatively higher activation in these areas during self pain) and activation in the aMCC (for which no difference was found across these conditions). Relatively higher activation of mPFC during other pain conditions compared to self pain conditions is also broadly consistent with a noted role for this region in empathy but not personal distress or experience of pain in the self. The model additionally makes some predictions relating to the two mPFC-mediated caregiving pathways, which are a result of the weights we have chosen. In particular, our model predicts that both of the mPFC-mediated caregiving pathways (mPFC–BLMA⊕–NAc/VP and mPFC–mPOA–VTA–NAc–VP) will be active during both weak and strong empathic concern states and that they will both increase activation across these phases (whereas it might be that, for example, activation in the mPFC–mPOA–VTA–NAc pathway is relatively stable across these phases, with increases in caregiving output in the strong empathic concern state mediated mostly by increases in stimulation of the VP by the BLMA). According to the model, mPOA and VTA activation will be highest in the strong empathic concern state relative to weak empathic concern, with mPOA and VTA firing rates roughly equal and greater than VP firing rates, which are in turn greater than those for the NAc, across both states. During the personal distress state, mPOA firing rates are predicted to be too low to result in any significant firing in the VTA and NAc (as opposed to activation along this pathway, but insufficient BLMA⊕ stimulation of NAc to release VP from inhibition, which could result in similar caregiving output). These predictions can potentially be tested empirically (e.g., in empathic scenarios involving a behavioral response in which stimuli depicting self, close other, and distant other are used with the aim of differentially activating the vmPFC), and weights in the model can be tuned accordingly to account for any discrepancies.

## A MODEL OF THE SELF-ATTACHMENT EMPATHY PROTOCOLS

Up to this point, we have considered states of personal distress and (weak and strong) empathic concern within an individual (e.g., a psychotherapist) resonating with the negatively valenced emotional state of another (e.g., the client) and have shown how these states are mediated by the relative strength of self–other emotional state attribution along with the perceived degree of self-relatedness of the other. We now extend this model to consider self-attachment therapy, in which the individual takes the role of both psychotherapist and client. In particular, we propose a hypothetical model of how self-attachment therapy might gradually progress from a state of personal distress to one of strong empathic concern (as the conceptualization stage is completed) to one of compassion (as a result of empathically motivated self-directed bonding) in which inner child–directed caregiving behavior is mediated by a positive (compassionate) rather than negative (empathic) emotional state.

### Bonding Protocols

Before detailing our proposal regarding the hypothesized effects of empathic resonance with the inner child, we briefly review our previously presented neurobiological hypothesis with regard to the effects of the self-attachment bonding protocols (Cittern, 2017; Cittern & Edalat, 2015) that this empathic concern is aiming to motivate as behavioral output. The bonding protocols are concerned with the individual forming an imaginative but passionate affective bond with the inner child (from the perspective of the adult self), which is subjectively experienced as falling in love with him or her. The bond-making process can be further enhanced with the use of activities like self-massage and (overt and/or imagined) song and dance directed toward the inner child, which are proposed to stimulate reward circuitry.

At a neural level, the internal working model (attachment schema) has been theorized to be based in unconscious and implicit memories, rooted mainly in right hemisphere (RH) brain regions centered on the OFC, amygdala, and hypothalamus (Cozolino, 2006; Schore, 2003, p. 139)—areas known to be central to, and crucially involved in, a broad range of social cognition and emotional processing functions. Largely mature at birth (Ulfig, Setzer, & Bohl, 2003), the amygdala is crucially involved in fear conditioning (Milad & Quirk, 2012), saliency, and stress-related processes that are likely to underpin many forms of insecure (particularly disorganized) attachment (Main & Hesse, 1990). High levels of attachment anxiety have been found to correlate with elevated cortisol profiles (Kidd, Hamer, & Steptoe, 2013) and a relatively overactive amygdala in response to angry faces conveying negative social feedback (Vrtička, Andersson, Grandjean, Sander, & Vuilleumier, 2008) and infant crying (Riem, Bakermans-Kranenburg, van IJzendoorn, Out, & Rombouts, 2012).

The amygdala has strong bidirectional connectivity with the OFC, which represents many types of primary reward, including positive tactile stimulation (Rolls, 2013), and parts of which have been found to preferentially activate in mothers viewing images of their own as opposed to others’ infants (with activation levels correlating with self-reported pleasant mood ratings; Minagawa-Kawai et al., 2009; Nitschke et al., 2004), with medial regions crucially involved in the learning of stimulus–reward associations (Walton, Behrens, Buckley, Rudebeck, & Rushworth, 2010). The OFC is believed to mediate stress and facilitative reactivity to social stimuli via projections to the dorsomedial hypothalamus (dmH) and the paraventricular nucleus of the hypothalamus (PVN). The parvocellular part of the paraventricular nucleus of the hypothalamus (PVNp) releases corticotropin-releasing hormone (CRH; the precursor to stress hormone cortisol), which stimulates stress circuitry focused on the central nucleus of the amygdala (CeA; a major output nucleus of the amygdala involved in processing pain and fear responses; Zimmerman, Rabinak, McLachlan, & Maren, 2007) and the locus coeruleus (LC; which is involved particularly in arousal and alertness aspects; Benarroch, 2009), while the magnocellular part of the paraventricular nucleus of the hypo thalamus (PVNm) releases OXT, one effect of which is thought to be a modulation of DA release (Love, 2014). Evidence implicates both DA (Bartels & Zeki, 2004; Strathearn, Fonagy, Amico, & Montague, 2009; Vrtička et al., 2008) and OXT (Feldman, Weller, Zagoory-Sharon, & Levine, 2007; Gordon, Zagoory-Sharon, Leckman, & Feldman, 2010) as being crucial for a range of bonding and attachment-related behaviors within circuitry involving the vmPFC, amygdala, and hypothalamus (Atzil et al., 2017). However, exogenous OXT administration has in certain cases been found to increase antisocial tendencies (Bartz et al., 2011; Declerck, Boone, & Kiyonari, 2010) and appears to amplify preexisting interpersonal schemas (Bartz et al., 2010; Olff et al., 2013), making it unsuitable as a treatment for many attachment-related disorders.

In light of the preceding, we have previously hypothesized (Cittern, 2017; Cittern & Edalat, 2015) that a main effect of the bonding protocols is to associate broad classes of social stimuli that have previously been conditioned as being fearful or threatening in nature with representations of additional, naturally induced rewards (which result from various interactions, e.g., self-massage or directed singing, Jeffries et al., 2003; Kleber et al., 2007; Salimpoor, Benovoy, Larcher, Dagher, & Zatorre, 2011, with inner child imagery, Strathearn, Li, Fonagy, & Montague, 2008). At a neural level, we proposed that these new stimulus–reward associations in mOFC would result in a rebalancing in activation of stress and facilitative circuitry in response to such classes of social stimuli. As the bonding protocols progress, the mOFC should gradually come to learn new reward associations, increasingly facilitating natural endogenous OXT release and inhibiting CRH release, while dopaminergic reward-prediction errors should drive a vmPFC-mediated inhibition of stress circuitry via strengthening of an intercalated cells of the amygdala (ITC)–CeA pathway, inhibiting activity in the amygdala and stress circuitry.

#### Bonding protocols and compassionate states

The self-attachment bonding protocols are closely related to the concept of compassion. In a compassionate state, as for an empathic state, there is a strong self–other distinction along with knowledge of the emotional state of another (Gonzalez-Liencres et al., 2013). Also in similarity with an empathic state, a compassionate state in the self can motivate prosocial behavior aimed at relieving a perceived negative state in the other. The key distinction between empathic and compassionate states is that compassionate states do not necessarily involve the mirroring of the emotional state of the other. For example, one may perceive suffering in another, but, rather than mirroring this suffering and experiencing this within the self (as in an empathic state), a compassionate state would instead involve positively valenced emotion within the self. In a compassionate state, it is this positively valenced emotion within the self, rather than negatively valenced emotion that is mirrored from the other, that can motivate prosocial behavior aimed at alleviating the suffering of the other. With respect to self-attachment, our proposal is that application of the bonding protocols toward the conceptualized inner child serves to engender a more compassionate stance within the adult self.

Whereas empathy-for-pain states have been found to activate overlapping regions involved in self pain, compassion instead seems to activate areas associated more with love, reward, and positive emotion. For example, Klimecki, Leiberg, Ricard, and Singer (2013) investigated subjective emotional states and neural activations in response to another’s distress, before and after both short-term empathy and compassion training. Empathy training increased empathic responses and negative affect and was associated with activation in core AI–aMCC empathy-for-pain circuitry. Following compassion training (which involved watching videos of others in distress and cultivating feelings of benevolence toward them), negative affect response to others’ pain returned to baseline, while activation in areas associated with positive affect (including mOFC, reward circuitry in the ventral striatum [which includes the NAc], and perigenual ACC) increased. A related study examined neural activations involved in the formation of a compassionate state in response to someone in distress, as opposed to a reappraisal (i.e., down-modulation of negative affect; Engen & Singer, 2015), with compassion compared to both reappraisal and passive watching of the same negative stimuli resulting in increased activation in vmPFC, mOFC, perigenual ACC, and NAc.

### Self-Attachment Empathy Protocols: A Hypothesis

On the basis of our model of personal distress and (weak and strong) empathic concern states, we now attempt to form a hypothesis with respect to how the self-attachment empathy protocols might result in neural activation corresponding to a strong empathic concern state toward the inner child and how repetitive application of the self-directed caregiving and bonding (which is proposed to result as motivated behavior from this strong empathic concern) might in turn facilitate a gradual shift toward a pattern of network activation corresponding more to a compassionate state within the adult self.

In particular, we propose that a sufficient self–other distinction is required for the mPFC to stimulate the mPOA–VTA–NAc–VP, BLMA–VP, and BLMA–NAc–VP pathways (as described in Numan’s model) that facilitate caregiving behavior. Recall that there is evidence to sug gest the vmPFC encodes “close other” representations with high self-relatedness (Denny et al., 2012) and that ventral parts of the mPFC have been found to activate more strongly within the context of empathy with respect to the perceived closeness (Meyer et al., 2012) and innocence (Fehse et al., 2015) of the other. Thus, in line with Numan’s proposal that more ventral parts of the mPFC might be involved in empathic concern, we suggest that stimulation of the caregiving pathways will be strongest for mPFC neural populations that encode a “close other” that is perceived as having high self-relatedness/similarity and innocence (located in vmPFC), which are precisely the characteristics possessed by the conceptualized inner child within the self-attachment framework. Within the context of empathically motivated care giving behavior, this corresponds to the strong empathic concern state that we detailed earlier. Under the alternative view of mPFC function (proposing that ventral/dorsal areas encode executed/modeled value; Nicolle et al., 2012) discussed previously, as the adult self and inner child distinction is developed (and the idea of tending to the distressed inner child is formulated), we might similarly expect progression toward patterns of activation (in more ventral areas) representing a high value of caregiving behavior (executed by the adult self) for the inner child.

As discussed previously, interactions between the mPOA and the mesolimbic DA system, along with increased functional connectivity between the amygdala and NAc and VP, have been observed during the simulation of prosocial acts. In terms of self-attachment therapy, this suggests that we might expect the mPOA–VP and BLMA–VP pathways to be activated both when the adult self imagines, and overtly practices, caregiving and bonding behaviors with the inner child. As we have overviewed, we previously hypothesized that one effect of the self-attachment bonding protocols would be new attachment-related stimulus–reward associations in the mOFC (an area that represents many forms of primary reward and is involved in learning stimulus–reward associations; Rolls, 2013; is known to increase firing in response to stimuli that predict rewards with activation levels that are correlated with reward value, Gottfried, O’Doherty, & Dolan, 2003; and has been associated with positive emotional states and compassionate stances, with heightened activation found in this area following compassion training in human subjects; Klimecki et al., 2013). Thus, within the context of our model of empathically motivated caregiving behavior here, we hypothesize that with repetitive application of the bonding protocols, mOFC activation should increase in response to the distressed inner child stimulus. In this way, the mOFC should be expected to increasingly stimulate both inhibitory BLMAi (which inhibit the negatively valent BLMA⊖ neurons) and positively valent BLMA⊕, resulting in caregiving behavior that gradually comes to be facilitated via a positive (OFC-mediated) rather than negative (BLMA⊖-mediated) emotional state.

### Additional Regions and Connectivity

**Figure 8.**
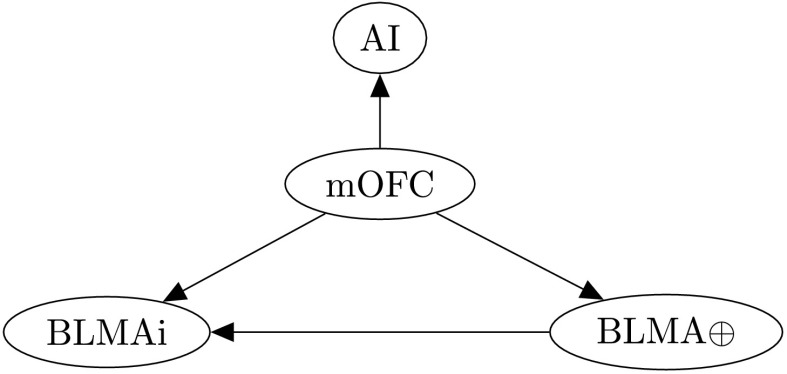
**Additional connectivity emanating from the mOFC to the AI, and BLMAi and BLMA***⊕*****.The remaining architecture is as in Figure 3.

In his model of empathically motivated caregiving behavior, Numan proposes OFC projections to the AI and BLMA as additional pathways by which caregiving behavior might be modulated by in-group preferences (Numan, 2014, p. 279). On the basis of the previously proposed role for the OFC in the self-attachment bonding protocols, along with findings from the neuroscience of compassion, we extend our model to incorporate projections from mOFC to these regions (Figure 8), which are proposed to increase in strength as the bonding protocols (Stage 3) progress. Details of the number of neurons, neuron types and target connection probabilities and weights for this additional neural group and its synapses are given in Table A.9.

### Desired Network States and Simulation Phases

As described previously, the empathy protocols involve focusing on an image of oneself (the inner child) as an infant/child in distress and empathizing with the inner child to motivate caregiving in the form of the bonding protocols. Here we describe three network states that are modeled in distinct phases of our simulation. On the basis of our previously proposed model of personal distress and (weak and strong) empathic concern, we suggest that a typical individual undertaking self-attachment therapy might progress through these phases sequentially as the protocols are undertaken, and the simulations that follow thus provide predictions for patterns of activity that might be hypothesized to occur (under the assumption that the previously proposed model of personal distress and empathic concern states holds and that the therapy induces the patterns of injected current that are to be described across each phase). Stimulation of the BLMA⊖ (now corresponding to a focus on the stimulus representing the distressed inner child) across the length of the simulation is as before, but note that we now have an additional neural group (mOFC) that receives different amounts of current injection at each time step (to stimulate different activation levels that are hypothesised to correspond to varying levels of progression with the bonding protocols).

#### Personal distress

Initially, on application of the empathy protocols, it may be that there is only a weak distinction between the self (adult self) and other (inner child), that is, high activity in the self pain network and low activity in the other pain network. Thus the first state that we want to capture is that of personal distress within the adult self. In this state, the adult self focuses on the image of the inner child and mirrors his or her negative emotional state, but the lack of self–other distinction might be expected to result in personal distress: In accordance with the findings of Ochsner et al. (2008) described previously, AI and PI activations should be relatively high for this state, which involves a form of self pain. Because personal distress is associated with withdrawal rather than prosocial behavior, we should additionally have low activity in the VP (which drives caregiving behavior). Relatively high activity in the BLMA⊖–AI–aMCC network, along with low activity in the VP, thus defines a network state hypothesized to correspond to personal distress during the early stages of self-attachment.

Current injections for the first simulation phase (*t* ∈ (0,10] s) are as previously defined for the personal distress state, but now have an additional stimulation of the mOFC (to represent low levels of reward association with the inner child stimulus, i.e., weak progress with respect to the bonding protocols in Stage 3 of the therapy). This corresponds to stimulating random neuron subsets *c*_*t*_(mOFC) ⊆ *g*_*t*_(mOFC) (with $\left|c_{t}(\text{mOFC})|∼U\{|\text{mOFC}|*L,|\text{mOFC}|*M\right\}$).

#### Strong empathic concern with strengthening conceptualization

The second state that we want to capture is an empathic state with a sufficiently strong self–other distinction such that caregiving (i.e., the self-directed bonding in Stage 3 of the therapy) results as motivated behavior. During this state of strong empathic concern, we should again have activation in the BLMA⊖ (stimulated as a result of focusing on the inner child stimulus) and AI and aMCC (which form core parts of empathy circuitry). In accordance with the findings in Ochsner et al. (2008), AI and PI activations should be relatively low compared to a personal distress state, while aMCC should be roughly the same. In this state, activity in mPFC neurons encoding other-referential representations should trigger activity in the BLMA⊕–VP and mPOA–VTA–NAc–VP pathways that facilitate caregiving behavior.

In particular, during this phase of the simulation, we want to capture a transition from personal distress (with weak self–other distinction) to strong empathic concern (with a well-developed self–other distinction and a close other representation), which is proposed to occur as conceptualization of the inner child (Stage 2 of the therapy) progresses. This transition is assumed to correspond to a linear shift in input current into the insular, aMCC, and mPFC, representing a gradual shift between the states of personal distress and strong empathic concern (that we previously defined) that might occur as the concept of the inner child as a distinct entity is developed. Thus, in addition to current injections into the BLMA⊖, we also inject currents into a proportion of neurons in the AI and PI that linearly decreases from “high” to “low” as the second phase (*t* ∈ (10,20] s) progresses. This corresponds to stimulating a random subset *c*_*t*_(AI) ⊆ *g*_*t*_(AI) of AI neurons (with |*c*_*t*_(AI)| = ((|AI|* (*L* − *H*)) * (*t*/*p*)) + |AI|* (2 * *H* − *L*)), and a random subset *c*_*t*_(PI) ⊆ *g*_*t*_(PI) of PI neurons (with |*c*_*t*_(PI)| = ((|PI|* (*L* − *H*)) * (*t*/*p*)) + |PI|* (2 * *H* − *L*)). To capture increasing activation in the other pain network, we inject currents into a proportion of neurons in the aMCC and mPFC that linearly increases from “low” to “high” as a function of phase progression. This corresponds to stimulating a random subset *c*_*t*_(aMCC) ⊆ *g*_*t*_(aMCC) of aMCC neurons (with |*c*_*t*_(aMCC)| = ((|aMCC|* (*H* − *L*)) * (*t*/*p*)) + |aMCC|* (2 * *L* − *H*)) and a random subset *c*_*t*_(mPFC) ⊆ *g*_*t*_(mPFC) of mPFC neurons (with |*c*_*t*_(mPFC)| = ((|mPFC|* (*H* − *L*)) * (*t*/*p*)) + |mPFC|* (2 * *L* − *H*)).

The retrieval of autobiographical memories of familiar others involves activation of regions including the precuneus and PCC (Maddock et al., 2001), which are both regions in Zaki’s other pain network. One possibility is that conceptualization protocols involving the individual actively attempting to associate an image of his or her younger self in distress with the conceptualized inner child “other” result in plasticity in circuits involving these regions, which might in turn account for a change in activation in self and other pain network activity (modeled here in terms of a simple linear shift in current injection). As we discuss in Summary and Future Work, OXT (hypothesized to be released as a result of the bonding protocols) may also play a role in facilitating this self–other shift. Thus the neural mechanisms underlying this transformation are likely to be complex and multifaceted and to differ according to the precise conceptualization techniques that are employed, and we leave the further detail for future work.

As in the first phase, current is injected into the mOFC at low levels (to induce levels of activation corresponding to network states before which application of the bonding protocols have begun to take effect) with stimulation of a random subset *c*_*t*_(mOFC) ⊆ *g*_*t*_(mOFC) of mOFC neurons (with $\left|c_{t}(\text{mOFC})|∼U\{|\text{mOFC}|*L,|\text{mOFC}|*M\right\}$).

#### Compassion

The third network state that we want to capture corresponds to a more compassionate state, which occurs as a result of effective application of the bonding protocols (Stage 3) and consequentially updated stimulus–reward associations in the mOFC. Now when the adult self focuses on the distressed image of the inner child, instead of mirroring the negative affective state, we propose that the adult self will instead have a positive (compassionate) inner emotional state and that this state will continue to motivate prosocial bonding behavior toward the inner child.

As discussed previously, we have hypothesized that one effect of the application of the self-attachment bonding protocols is to stimulate activity in neurons of the OFC and vmPFC representing positive reward and emotion, which in turn inhibit negatively valent representations in the amygdala. In our model of the empathy protocols here, this effect corresponds to stimulation of BLMA⊕ by the mPFC (which occurs during both empathic concern and compassionate states) and mOFC (which occurs uniquely during compassionate states) and stimulation of BLMAi by mOFC, which in turn inhibits BLMA⊖ (which again occurs uniquely during a compassionate state). The mOFC also stimulates AI, which is consistent with evidence suggesting a role for the left AI in positive emotion and maternal behavior (Craig, 2009 Figure 2). Stimulation of BLMA⊕ and a different (presumed positively valent) subpopulation of AI neurons, along with inhibition of BLMA⊖, is proposed to represent neurally the positively valent emotional elements of a compassionate state within the adult self. This state should also result in continued activation in Numan’s caregiving pathways, facilitating bonding behavioral responses toward the inner child.

During the third phase (*t* ∈ (20,30] s), in addition to current injections into the BLMA⊖, we also inject currents into a “low” proportion of neurons in the AI and PI, to represent low levels of activity in the self pain network. This corresponds to stimulating a random subset *c*_*t*_(AI) ⊆ *g*_*t*_(AI) of AI neurons (with $\left|c_{t}(\text{AI})|∼U\{|\text{AI}|*L,|\text{AI}|*M\right\}$) and a random subset *c*_*t*_(PI) ⊆ *g*_*t*_(PI) of PI neurons (with $\left|c_{t}(\text{PI})|∼U\{|\text{PI}|*L,|\text{PI}|*M\}\right)$. To capture a strong self–other distinction, it is also assumed that activity in the other pain network is sustained during this phase so that we inject current into a “high” proportion of aMCC and mPFC neurons. This corresponds to stimulating a random subset *c*_*t*_(aMCC) ⊆ *g*_*t*_(aMCC) of aMCC neurons (with $\left|c_{t}(\text{aMCC})|∼U\{|\text{aMCC}|*M,|\text{aMCC}|*H\}\right)$ and a random subset *c*_*t*_(mPFC) ⊆ *g*_*t*_(mPFC) of mPFC neurons (with $\left|c_{t}(\text{mPFC})|∼U\{|\text{mPFC}|*M,|\text{mPFC}|*H\}\right)$. To capture successful application of the bonding protocols, we linearly increase the proportion of mOFC neurons receiving input across the phase by stimulating a random subset *c*_*t*_(mOFC) ⊆ *g*_*t*_(mOFC) of mOFC neurons (with |*c*_*t*_(mOFC)| = ((|mOFC|* (*H* − *L*)) * (*t*/*p*)) + |mOFC|* (3 * *L* − 2 * *H*)). As we have discussed, increasing activation here is proposed to correspond to increasing levels of reward associated with the inner child stimulus as a result of this self-directed bonding.

### Simulation Results

**Figure 9.**
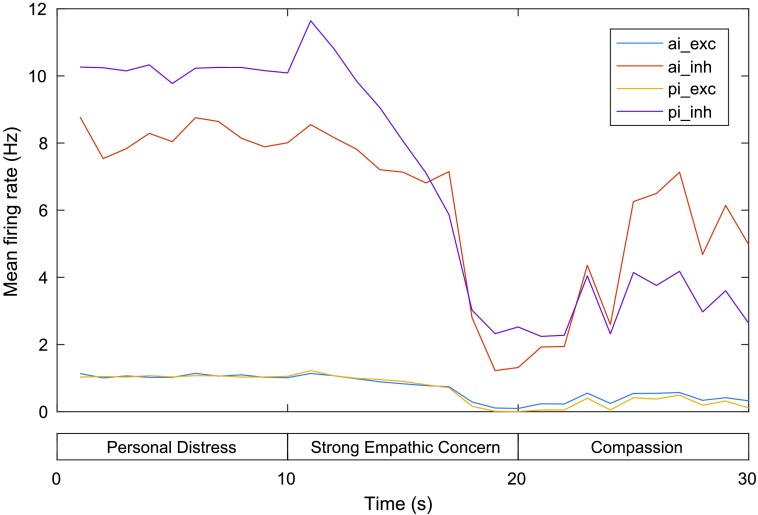
**Mean firing rates for the AI and PI in the model of the self-attachment empathy protocols.** exc = excitatory neurons, inh = inhibitory neurons. See text for details.

Figure 9 gives the MFR for neurons in the excitatory and inhibitory AI and PI neural groups. The chart shows that the MFR of AI and PI neurons is highest for the first phase of the protocol but drops significantly throughout the second phase (as self pain inputs are decreased and other pain inputs are increased), in accordance with the previously simulated personal distress and strong empathic concern states. Excitatory/inhibitory AI neurons drop from a MFR of 1.01/8.01 Hz at 10 s (end of the first phase and beginning of the second phase) to 0.1/1.31 Hz at 20 s (the end of the second phase), while excitatory/inhibitory PI neurons drop from a MFR of 1.05/10.09 at 10 s to 0.01/2.52 at 20 s. Firing rates for the AI rise again slightly during the third phase to 0.32/4.97 at 30 s owing to mOFC input, in line with a role for this region (particularly in the left hemisphere) in positive emotion and maternal behavior (outlined previously).

**Figure 10.**
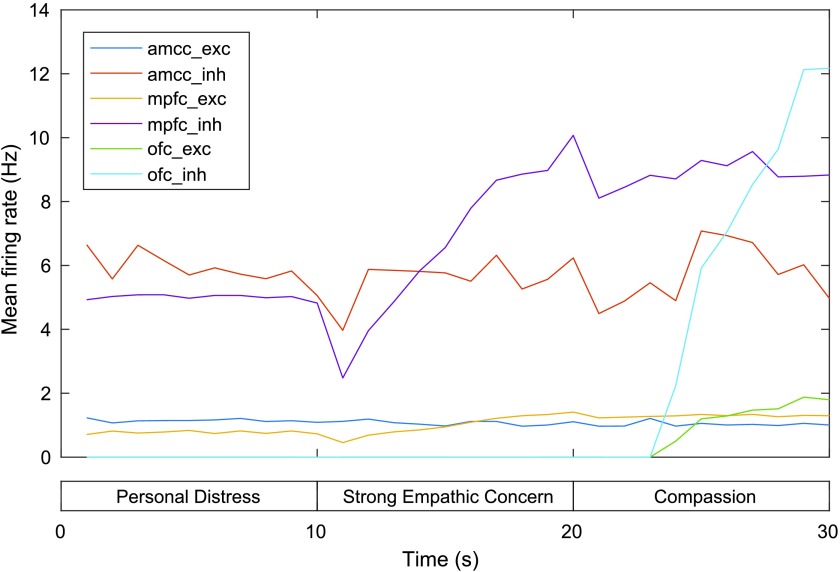
**Mean firing rates for the aMCC, mPFC, and mOFC in the model of the self-attachment empathy protocols.** exc = excitatory neurons, inh = inhibitory neurons. See text for details.

MFR for neurons in the excitatory and inhibitory aMCC, mPFC, and mOFC groups are shown in Figure 10. The aMCC MFR is relatively flat across the three phases: The MFR for excitatory/inhibitory aMCC is 1.09/5.06 Hz at 10 s, 1.11/6.23 Hz at 20 s, and 1.01/4.97 Hz at 30 s. Stability across the first and second phases mirrors the previously simulated personal distress and strong empathic concern states and is in accordance with the findings of the experiments on self and other pain attribution (described previously), which did not find significant differences in activation of the aMCC across self and other pain paradigms. The relative decrease in firing rate for the aMCC during the third phase is consistent with evidence (discussed previously) that different (more perigenual) areas of the ACC may instead be involved in compassionate states.

The MFR of the mPFC rises steadily during Phase 2 (from 0.73/4.82 Hz at 10 s to 1.41/10.07 Hz at 20 s) following relative stability in the first phase and back toward relative stability in the third phase. The rise in mPFC activation coincides with a strengthening of the self–other distinction between adult self and inner child, which is captured by increasing activation of (more ventral) neurons encoding close other representations proposed to be associated with the inner child. For the mOFC, the MFR is relatively low during the first and second phases but rises as the third phase progresses (with the MFR for excitatory/inhibitory mOFC neurons rising from near zero at 20 s to 1.80/12.17 Hz at 30 s). This rise during the third phase corresponds to progress in the application of the bonding protocols with respect to increasing expectations of reward associated with prosocial motivation toward the inner child.

**Figure 11.**
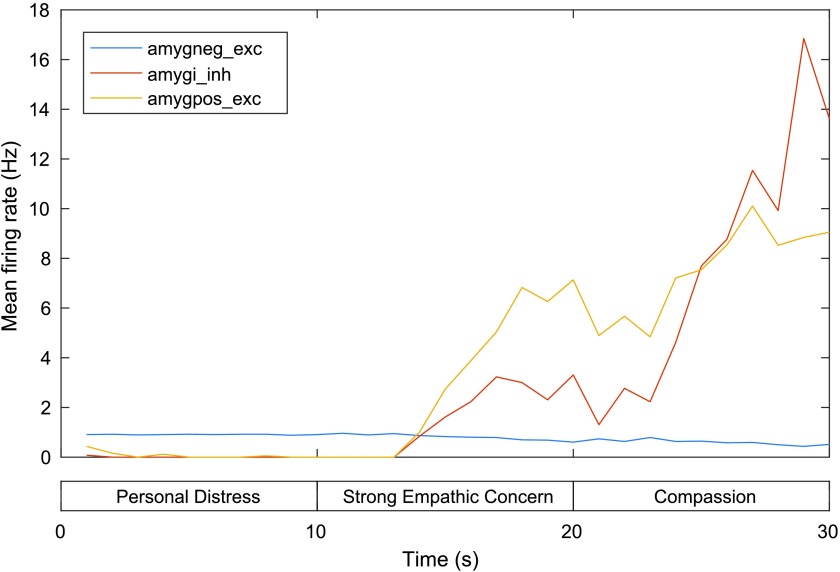
**Mean firing rates for the three BLMA neural populations in the model of the self-attachment empathy protocols.** exc = excitatory neurons, inh = inhibitory neurons. See text for details.

Figure 11 shows the MFR for the three BLMA neural populations. The BLMA⊖, which receives a steady input current across all three phases (corresponding to inner child stimulus input) has a MFR that is relatively flat during the first phase (0.91 Hz at 10 s), but drops as the self–other distinction becomes stronger during the second phase (to 0.61 Hz at 20 s) and throughout progression of the third phase (to 0.51 Hz at 30 s). This drop during the third phase coincides with a relatively large increase in MFR of the BLMAi (from 3.31 Hz at 20 s to 13.62 Hz at 30 s), whose neurons are stimulated by the mOFC. In contrast, the MFR of the BLMA⊕ is relatively low during the first phase but rises for strong self–other distinction at the end of the second phase (to 7.13 Hz) and again as the third phase progresses (to 9.05 Hz at 30 s). During the first and second phases, then, we have relatively low activation in the mOFC, BLMA⊕, and BLMAi and relatively high activation in the BLMA⊖, which, along with activation in the AI and aMCC, is proposed to correspond to the negatively valenced emotional state within the adult self that is mirrored from the inner child. During the third phase, the MFR in the BLMA⊖ falls significantly, yet rises in the BLMA⊕ and mOFC, and the mOFC stimulates distinct neurons in the AI. We propose that this pattern of activation corresponds to a positively valenced compassionate state within the adult self, in which the negatively valenced, empathically mirrored emotional state of the previous phases is (at least somewhat) suppressed.

**Figure 12.**
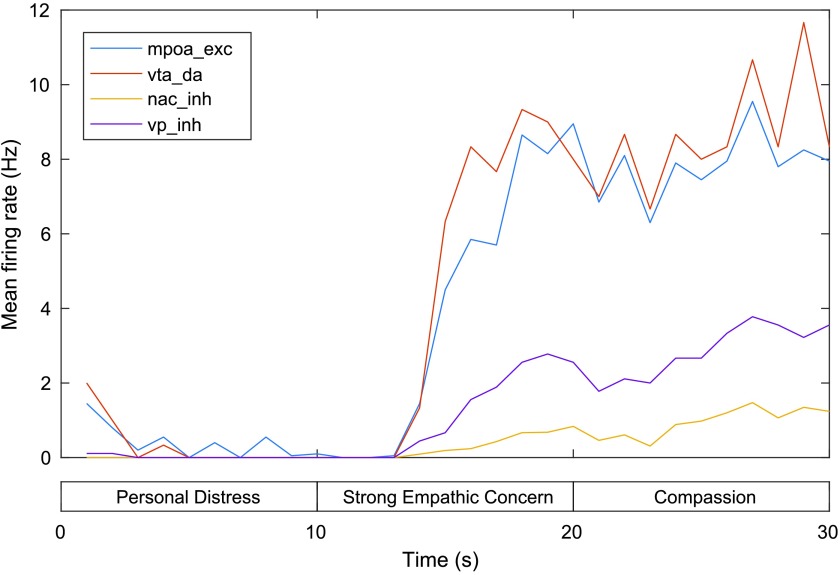
**Mean firing rates for the mPOA, VTA, NAc, and VP in the model of the self-attachment empathy protocols.** exc = excitatory neurons, inh = inhibitory neurons, da = dopaminergic neurons. See text for details.

MFR for the mPOA, VTA, NAc, and VP are shown in Figure 12. Activity in the mPOA, VTA, and NAc is relatively low during the first phase and rises significantly toward the end of the second phase (as the self–other distinction becomes stronger) and slightly further across the third phase. These firing patterns are reflected in firing in the VP, with relatively low MFR in the VP during the first phase (and no firing at 10 s), increased firing at the end of the second phase (with 2.56 Hz at 20 s), and relatively high (and rising) MFR as the third phase progresses (to 3.56 Hz at 30 s). An increasing MFR for the VP as the second phase progresses represents increasing facilitation of caregiving behavior in response to a strong empathic concern state, as a result of strengthening self–other distinction. The MFR for the VP is highest toward the end of the third phase, which corresponds to an increase in caregiving behavior as the internal state of the adult self transitions from an empathic state toward a more compassionate one.

In summary, our biologically plausible model replicates existing data on relative AI and PI activation across self and other pain states (with relatively higher activation in these areas during self pain), activation in the aMCC (for which no difference was found across self and other pain conditions), and relative activation in the mOFC in compassion compared to noncompassion (strong empathic concern and personal distress) states, resulting in appropriate output in caregiving pathways across these distinct states. Our model additionally makes a number of new predictions, which are partly a result of the particular weights that we have chosen in our simulations. These include that activation in mPFC neural populations will be relatively stable across strong empathic concern and compassion stages; that both mPFC-mediated caregiving pathways will be active during strong empathic concern and compassion, with rising activation across these states; and that inhibition on negatively valent BLMA⊖ neurons mediating avoidance will be higher in compassion as compared to strong empathic concern states. As with those for the model of personal distress and empathic concern, these predictions can potentially be tested (with suitably designed experimental scenarios) and the model refined accordingly, as can our hypotheses related to self-attachment therapy (e.g., that inner child representations will activate those vmPFC neurons encoding close/innocent others and that mOFC-mediated inhibition of BLMA⊖ will occur as a result of self-directed bonding).

## SUMMARY AND FUTURE WORK

On the basis of an existing neuroanatomical model of how empathic states can motivate caregiving behavior, fMRI data on self- and other-referential processing, and networks involved in the perception of pain in self and others, we presented a spiking neural model capable of describing three distinct empathy-related states: personal distress (involving emotional attunement within the context of a weak self–other distinction), weak empathic concern (prosocial motivation arising from emotional attunement, a strong self–other distinction, and percep tions of a relatively distant other), and strong empathic concern (empathically motivated prosocial behavior arising from representations of a relatively close other). We then extended this model to the case of self-attachment therapy: an attachment-based psychotherapy which involves a conceptualized adult self empathizing with an inner child to motivate bonding behavior toward him or her. We used this model to present a hypothesis as to how self-attachment might facilitate a transition within the adult self from a state of personal distress to one of strong empathic concern to a compassionate stance toward the inner child as the therapy progresses.

One of the main considerations for any modeling effort is the selection of an appropriate level of abstraction. Because our hypotheses on the effects of self-attachment therapy have to date been formulated based on existing related data on firing and plasticity in particular brain regions, we chose to use a relatively detailed and biologically plausible spiking neural model. Such an approach clearly has limitations owing to the resulting complexity of the model, for example, in terms of the large parameter space for connectivity weights and the necessity to compartmentalize (and ignore regional interactions that might in future be determined to be significant for explaining the emergent properties of interest). Nonetheless, this method has certain advantages with respect to following an empirical hypothesis-testing approach to therapy development in that predictions for changes in regional plasticity and firing rates made by such models can be relatively easily and directly tested (e.g., using imaging). Indeed, our aim is for future versions of the model to be able to capture the initial conditions (connectivity) characterizing different groups of individuals (e.g., according to attachment type or prevalence of borderline symptoms). In this way, given a target network state corresponding to a secure attachment schema, testable predictions made by the model might directly inform development of self-attachment therapy (e.g., by determining which pathways, and thus intervention methods, might be most effectively targeted in each case). The work presented here is a small step in that direction, and we discuss now some additional ways in which the model’s biological accuracy might be improved in future iterations.

The first point to note is that we did not consider a full realization of self and other pain networks but rather argued that current injection into the insular, aMCC, and mPFC could feasibly represent activity in these two networks. Our model can thus be expanded to capture in more detail networks facilitating the perception of pain in self and other as these data become available and also incorporate additional regions (such as those in the temporal poles, and the temporoparietal junction, which includes parts of the STS along with the inferior parietal lobule) that are commonly implicated in studies investigating cognitive empathy, theory of mind, and mentalization (Abu-Akel & Shamay-Tsoory, 2011; Frith & Frith, 2006; Walter, 2012). Furthermore, we considered only anterior midcingulate parts of the ACC across empathy and compassion phases, whereas evidence (discussed earlier) suggests that more perigenual areas of the ACC are involved in compassionate states.

With respect to the regions that we did consider, our model is still highly simplified. Although all of our estimates for number of neurons were based on nonclinical human data, some were inaccurate owing to lack of finer data (in particular, the mPOA, for which we used the volume of the whole encapsulating anterior-superior hypothalamic region; the PI, where we used a neural density for adjacent AI; and the NAc, for which we used an estimate for the total number of neurons despite the caregiving pathway likely involving neurons more in the shell region). Furthermore, we defaulted to regular-spiking (for mPOA), fast-spiking (for VP), and intrinsically bursting (for VTA) neuron types in the absence of more detailed models and typically only considered neuron types that either form a majority or have been proposed as crucially important in each region.

Owing to a lack of human data, we used animal data to define connectivity, although these data were not available in all cases. Accuracy of the model can thus be improved from this perspective as more connection data become available and also by considering spatial connectivity (Voges, Schüz, Aertsen, & Rotter, 2010) and potentially also laminar and columnar cortical structures. In addition, we did not consider connections between AI and NAc and between mPFC and NAc. These connections were proposed in Numan’s model to potentiate VP activation during empathic states, although details regarding axon terminals are for the time being unknown (Numan, 2014, p. 278): Future efforts can consider the nature of these additional connections. Finally, we assumed fixed connectivity weights with current-based synapses to demonstrate the three distinct states of the network, and so future work can consider conductance-based synapses and plasticity.

Studies that we highlighted have reported heightened AI activation with increasing perceptions of both closeness (Meyer et al., 2012) and in-group membership (Hein et al., 2010) of the other, and our strong (in contrast to weak) empathic concern state was broadly defined as involving representations of an other that was perceived as having these characteristics. Although our model replicated existing data on self and other perceptions of pain in the AI (with higher activation during the self pain condition), along with appropriate activation in the mPFC during weak and strong empathic concern states and appropriate subsequent activation in caregiving pathways in each case, activation in the AI across weak and strong empathic concern states did not significantly differ. Because (as we discussed) the mPFC is thought to be centrally involved in self–other representations (with more ventral parts encoding others with high self-relatedness, including closeness), and because Meyer et al. (2012) additionally reported increased functional connectivity between the mPFC and AI for an empathic response directed toward a close as opposed to distant other, it might be that this effect is mediated by mPFC projections to AI that involve higher connectivity from ventral (encoding close other representations) compared to more dorsal (encoding distant others) areas. Future work can thus attempt to improve the model in this regard.

As discussed, we previously hypothesised that one effect of the self-attachment bonding protocols is to stimulate OXT release from the PVNp, resulting in modulation of dopamine release in the VTA and enhanced vmPFC-ITC inhibition of the CeA and stress-related antisocial circuitry. In addition to this effect, we can predict that OXT release during the bonding protocols might enhance progress in the empathy protocols in a number of ways. As a result of known effects on receptors in the mPFC, mPOA, NAc, and BLMA, OXT release should in general potentiate caregiving (and suppress withdrawal) motivation during application of the empathy protocols (Numan, 2014, p. 287). In the case of the BLMA, we have considered the negatively valent input stimulus as directly stimulating BLMA⊖ in personal distress, empathic, and compassionate states (with indirect stimulation of BLMA⊕ occurring in empathic and compassionate states). However, OXT released during the bonding protocols might serve to facilitate additional and more direct stimulation of BLMA⊕ (and suppression of BLMA⊖ via stimulation of BLMAi, Numan, 2014). This means that we might expect the input stimulus to directly stimulate mostly BLMA⊖ neurons during personal distress but rather directly stimulate mostly BLMA⊕ neurons during the empathic and compassionate states.

OXT released as a result of application of the bonding protocols might aid in the transition from an empathic to a compassionately motivated state in response to viewing the negatively valenced inner child stimulus. We previously hypothesised that OXT-modulated DA would increase reward predictions and firing rates in the mOFC, but OXT release might moreover be involved in the suppression of activity in AI areas crucially involved in the formation of negatively valenced empathic states: Bos, Montoya, Hermans, Keysers, and van Honk (2015) found that empathy-related activation in the insular was strongly reduced after intranasal OXT in subjects observing others in pain. Furthermore, OXT release might serve to strengthen the self–other distinction. In Colonnello, Chen, Panksepp, and Heinrichs (2013), the ability of participants to differentiate their own identities was measured while they viewed photos of themselves morphing into photos of unfamiliar faces, with intranasal OXT shortening the time taken to differentiate self from other. These studies suggest a strong interdependence between the bonding and empathy protocols in self-attachment and point to a nature by which successful application of each is likely to drive progress in the other. Future work can consider in more detail the effects of OXT release on empathically motivated bonding, along with individual differences with regard to prior attachment experience. Because other types of human bonds (e.g., pair bonds) are thought to rely on overlapping circuitry between the amygdala, NAc, and VP, and because OXT and DA release into the NAc is thought to result in plasticity that enhances activation in NAc–VP circuitry that promotes such attractions, future work can also consider the potential implications for other types of human bonds (Numan & Young, 2016).

## ACKNOWLEDGMENTS

The authors thank Michael Numan for his valuable comments and suggestions and also the reviewers for their helpful feedback.

## AUTHOR CONTRIBUTIONS

Formulated project: DC, AE; devised and simulated model: DC; wrote the paper: DC, AE.

## Note

1 The authors used the term *compassion* to refer to a state more closely related to what we call *emotional empathy* and *sympathy*/*empathic concern*.
